# Proximity Iodo-labeling of Human Cervical Mucus with an Endogenous Peroxidase

**DOI:** 10.21203/rs.3.rs-11194466/v1

**Published:** 2026-10-06

**Authors:** John Tsibris

**Affiliations:** University of South Florida

**Keywords:** Human cervix, menstrual cycle, proximity labeling, heme peroxidases, proteomics

## Abstract

A calcium-dependent guaiacol peroxidase (GPx) was detected in the cervix and cervical mucus. Daily measurements of cervical mucus GPx can predict the day of ovulation by 4–5 days, since GPx activity drops significantly as 17beta-estradiol levels peak before ovulation. After ovulation, the concomitant rise of progesterone and GPx mark the end of the optimal time for conception. Cervical mucus was radiolabeled with hydrogen peroxide and Na125I, instead of guaiacol, to detect proteins iodinated by GPx in the follicular and luteal phases of the menstrual cycle. Based on the promising preliminary results, I describe the use of the non-radioactive Na127I and mass spectrometry to detect iodinated peptides and identify the autoiodinated GPx protein and its nearest neighbors. Among them enzymes and signaling proteins will help untie the Gordian knot of multicomponent protein complexes and transient interactions coordinated in the cervix during ovulatory menstrual cycles. Future studies could reveal whether different iodinated mucus proteins and their expression levels are associated with clinical symptoms and conditions during the menstrual cycle prompting research for new markers and therapy targets.

## Introduction

Human reproduction depends on the orchestration of hormones released during the menstrual cycle. A window to 17β-estradiol and progesterone levels in the peripheral blood was opened by monitoring the activity of guaiacol peroxidase, which in the human uterus is expressed only in the cervix^1^. Cervical mucus is produced in the endocervical epithelium by goblet epithelial cells^2,3^ and serves numerous functions in reproduction, notably as a barrier to impaired sperm. As ovulation time approaches, the precipitous drop of GPx levels correlates strongly with rising 17β-estradiol^4^ in synchrony with decreasing mucus viscosity and consistency that facilitate sperm penetration. The drop of the specific activity of GPx could be due to decreased GPx production and inactivation of secreted GPx by rising estrogens and is attributable only in part to increased water content of the mucus^4^. After ovulation, the sharp rise of serum progesterone is associated with the formation of thick mucus. GPx is increased in tandem suggesting that progesterone overcomes the effect of estrogens on GPx expression in the luteal phase. A mucus plug is formed if a pregnancy is achieved.

The specific activity of GPx during the menstrual cycle is shown on Fig. 1. The intriguing question is why some ovulating women have higher GPx levels than others. Perhaps, cycles with lower progesterone levels, indicating luteal insufficiency, would account for these differences following ovulation^4^.

Daily measurements of mucus GPx^6^ can define the optimal time for conception whether ovulation is early or late after the end of menses; the chronology of an individual’s menstrual cycles varies^7^. We noticed in a study volunteer that after the midcycle drop, her GPx remained high for the following 20 days because, as it turned out, she had unprotected intercourse during the test cycle and had become pregnant^4^. Another volunteer with normal hormone levels indicating ovulation contacted a severe vaginal yeast infection on the fifth day of sampling. We decided to treat her infection and continue with sampling. GPx levels remained high throughout her cycle arising, evidently, from peroxidases of activated lymphocytes, macrophages and neutrophils. Anovulatory cycles, spontaneous or secondary to contraceptive hormones, show high GPx throughout the cycle.

Cervical mucus fixation and dehydration introduce artifacts. Mucus in unfixed preovulatory samples is arranged in a dense filamentous structure with a net of interconnected fibers^8^. The average mesh size is 500 ± 250 nm, and the diameters of the fibers range between 10–500 nm. Ovulatory mucus is composed of floating globules with an average size of 600 ± 200 nm, that occasionally bridge together creating a short pearl necklace structure. In respiratory and intestinal mucus, mucin complexes promote disulfide-mediated polymerization to produce the mucus gel scaffold^8^. Proteomic studies of human cervical mucus have detected several members of the large family of heavily O-glycosylated mucins, MUC5B, MUC5AC, MUC6, MUC1, MUC4 and MUC16^9^. Myeloperoxidase is the only peroxidase detected in cervical mucus by proteomic analyses^10,11^.

Enzyme-catalyzed proximity labeling has emerged in recent years as a method to identify local proteomes, protein-protein, protein-RNA and protein-DNA interactions^12,13^. Briefly, the protein of interest (the bait) is coupled with an engineered tagging enzyme that covalently tags its neighbors with a peroxidase or biotin. Here, the yet unidentified cervical mucus peroxidase, which I refer to as “GPx”, is both the bait and the labeling peroxidase iodinating tyrosine residues of itself (autoiodination) and of its neighbors.

## Results

The results of the exploratory iodination experiment I ran in 1987 with cervical mucus from a study volunteer (Fig. 8^5^) are shown in Fig. 2. A nurse collected mucus samples at the external os after inserting a speculum. I iodinated samples collected on days 11, 15 and 17 after the onset of menses which was assigned as day 1. The iodinations were carried out with Na^**125**^I and H_2_O_2_ as described in the Methods. Na^**125**^I is a low-energy gamma emitter, with a 60-day half-life, used extensively and safely in bench research^14^. The radiograms are snapshots of the iodination mixtures fractionated by 2-dimentional isoelectric focusing and SDS-PAGE.

Figure 2 shows that “GPx” activity is present in the soluble and the particulate fraction of cervical mucus; a larger number of proteins was resolved on Day-15. In Fig. 2B, a sizable fraction of particulate proteins extracted with 0.5 M CaCl_2_ was degraded most likely by calcium-activated proteases. Very basic proteins such as myeloperoxidase (molecular mass, M_r_, 140 kDa and isoelectric point, pI, 9–10), lactoperoxidase (M_r_ 78 kDa, pI 9.2), and eosinophil peroxidase (M_r_ 66–71 kDa, pI 10.8) were outside the pH range of the isoelectric gel used. Deiodinases have very strict specificity^15,16^ and are unlikely to degrade the iodinated proteins in the absence of reductants.

## Methods

Cervical mucus is a non-homogeneous viscoelastic fluid. A sample of 20–400 mg was removed from the external os with a preweighed fertility cannula^6^; cotton adsorbs GPx irreversibly. Since freezing denatures the GPx activity of cervical mucus, we stored mucus at 4–8°C where it maintained activity for the few weeks I used it.

Iodination of cervical mucus at room temperature. Wet mucus (4–10 mg) was added to 10 mM Tris pH 7.2 and 5 mM CaCl_2_ at 100 μL/mg mucus, in a 1.5 mL Eppendorf tube and was dispersed by gentle stirring with a glass rod. Three μL of the Na^125^I solution (0.3 mCi) were added to the mixture and stirred gently for 10 min. Ten μL of 0.3% H_2_O_2_ were added per mL and the mixture stirred gently for 15 min. Another 10 μL H_2_O_2_ were added for 15 min. The reaction was stopped by freezing in acetone-dry ice bath, and the reaction mixture was dialyzed with tubing of 6–8,000 molecular weight cut-off in 10 mM Tris pH 7.2 for 3 hours changing dialysis buffer every hour. To the dialysate was added an equal volume of 20 mM Tris pH 7.2 and the dialysis continued overnight at 4°C. The dialysate was centrifuged at 100,000*g* for an hour and the supernatant solution, the soluble fraction, was dialyzed for 2 hours in 10 mM HEPES buffer pH 7.2 containing 10 mM calcium acetate changing the buffer after one hour and was stored at −20°C and used in the experiments. The pellet of the 100,000*g* centrifugation, the particulate fraction, was incubated overnight in 10 mM Tris pH 7.2 and 0.5 M CaCl_2_, centrifuged at 100,000*g* for an hour and the supernatant solution was further treated as the soluble fraction.

Isoelectric focusing and SDS-PAGE. The dialysates were fractionated with isoelectric focusing and SDS-PAGE gels followed by radiography.

## Discussion

Heme peroxidases defend against invading bacteria, fungi, parasites and viruses and catalyze a number of other reactions in the human cervix. Here, I describe the use of “GPx”, the unidentified peroxidase, to iodolabel proteins mainly at tyrosine residues and mass spectrometry to identify them and “GPx”. Marchalonis^17^ used lactoperoxidase from whey to radiolabel immunoglobulins and found that lactoperoxidase iodinated itself also; a synthetic peptide was iodinated at the tyrosine but not at its histidine residue. Could the soluble and the particulate autoiodinated protein “GPx” be a new peroxidase, an isoform of a peroxidase or a combination of peroxidases? Among the mammalian heme peroxidases likely to be “GPx” are myeloperoxidase, lactoperoxidase, thyroid peroxidase and eosinophil peroxidase^18^, but their levels are not known to correlate with sex steroid hormone levels during the menstrual cycle. GPx activity of endocervical epithelium is inhibited by estrogens^1^ and cervical mucus GPx correlates with estradiol and progesterone serum levels^4,6^. There are many estrogen-interacting proteins, such as protein disulphide isomerase (PDI) inhibited by estrogens in vitro^14^, and AGR2, an estrogen-responsive secreted PDI^19^ essential for the production of intestinal mucus, and a selective sensor of mucin misfolding as a non-canonical PDI^20^.

The zwitterionic non-denaturing detergent ASB-14^21^ should be tested to extract mucus while preserving protein-protein interactions. Specifically, cervical mucus is homogenized in 5 mM CaCl_2_ and ASB-14, centrifuged at 100,000*g* and the supernatant solution is used for various analyses. Also, cervical mucus can be homogenized in 5 mM CaCl_2_ and as shown in Methods, the soluble and particulate fraction, the latter extracted with ASB-14, studied separately. Although iodine-125 is not used in bench research now as in the past it could show expeditiously under what conditions of the redox environment^22^ in the soluble and particulate fractions the iodinating “GPx” might be rate-limiting as the iodination radius^13^ is extended. Iodine-125 could also facilitate tracing molecules, besides soluble proteins, which may be iodinated.

Since mucins contain relatively few tyrosine residues, the iodination radius could be adjusted to test if the abundant cervical mucins can be bypassed to label enzymes and other signaling proteins with higher number of tyrosine residues. In the mass spectrometric analysis, protein digestion is carried out from the N-terminus with trypsin and from the C-terminus with other reagents, such as GluC, to prevent also over digestion by trypsin of very basic proteins^21^ in cervical mucus. The detection of peptides with ^**127**^I-tyrosine can be made by adapting earlier proteomic methods^23–25^. The identification of “GPx” by mass spectrometry will be facilitated if there is no significant iodo-peptide background in the cervix since mammalian peroxidases share amino acid sequence similarities^18^. As an endogenous peroxidase, “GPx” and iodine, a small tag, are not expected to disrupt the structure of native cervical mucus but added H_2_O_2_ might unless used sparingly. As the iodination radius^13^ is extended, mass spectrometric analyses will map the spatial distribution of interacting iodinated proteins around “GPx”. Incubating cervical mucus with substrates, inhibitors and effectors of proximal enzymes may change the proximity labeling targets and point to structural reorganizations as they occur during the cycle.

Thyroid peroxidase (TPO) forms mucus plugs in airway epithelial cells, but not by its canonical reaction as a tyrosine iodinase^26^. TPO is not confined to the thyroid gland^26^, but has not been detected in the human cervix. In the biosynthesis of thyroid hormones, TPO is supplied with H_2_O_2_ by membrane-bound calcium-dependent DUOX2, the NADPH dual oxidase 2^27^. DUOX2 supplies H_2_O_2_ also to lactoperoxidase. Myeloperoxidase (MPO) catalyzes non-canonical reactions^28^, and recently a ground breaking study showed that a non-catalytic function of neutrophil MPO converts chromatin into an immune effector, independently of its peroxidase activity which is still active but uncoupled from its monomeric-dimeric structure^29^. MPO monomers and dimers have identical enzymatic activity^29^, which may prove relevant to the activity of soluble and particulate “GPx” on Fig. 2. Note that mitiperstat is an irreversible inhibitor of MPO. The discovery of a non-canonical tricarboxylic acid cycle in normal cells^30^ points to an intriguing metabolic mechanism that may operate in the cervix.

Great advances in proteomic strategies allow the identification of endogenous protein-protein interactions by a variety of analytical methods including proximity labeling^31^. In HEK293 cell cultures, APEX peroxidase labeled preferentially proteins in metabolic pathways within a 20 nm radius of the enzyme, whereas biotin-ligase BioID favored membrane proteins within a 10 nm radius^32^. Does the iodination of cervical mucus by the endogenous “GPx” have directionality^32^, for example, to intrinsically disordered proteins and regions? Disordered protein structural ensembles can be predicted from the amino acid sequence with STARLINK^33^.

Novel methods in cancer research with mucin-selective proteases^34^ could be used to degrade one of the mucins in cervical mucus samples and determine its effect on the iodination reaction. Removal of select mucins may provide insights about the mucus scaffold around “GPx” and the potential catalytic role of “GPx” to support the functions of mucus during the menstrual cycle.

A supplemental list of proximity enzymes and signaling proteins in cervical mucus can be obtained by analyzing also the endocervical epithelium from unfrozen and frozen hysterectomy tissues^1^. Samples of endocervical epithelium are extracted in 5 mM CaCl_2_ with ASB-14^21^, spun at 100,000*g* and the supernatant solution used in immunoprecipitations of key proteins from the iodination lists to detect their partners; western blots^21^ will confirm which partners are exported in cervical mucus during the cycle. The comprehensive list of proteins could be used to discover sentinel proteins in cervical mucus for various clinical presentations. Whole mount immunofluorescence can show protein-protein interactions in 3D^21^ in the vascular structure of the cervical epithelium.

There is increasing interest in cervical mucus exosomes^35^. Vaginal bacteria-derived extracellular vesicles, with a 50–250 nm diameter, can penetrate the luteal phase cervical mucus barrier whereas bacteria cannot^36^. In cancer, mitochondria traffic reprograms the metabolism of recipient cells^37^; mitochondria traffic may occur in the cervix during the menstrual cycle, in pregnancy and labor. The discovery that hormones influence whole-brain structural dynamics across the menstrual cycle^38^, adds the ultimate dimension to research for molecular communications during the menstrual cycle.

Expanding knowledge of tissue-resident macrophages^21,39–41^ poses many questions. Does “GPx” reside in macrophages of the human cervix, and as ovulation occurs with weeks-long regularity could metabolic economy dictate that groups of proteins be stored in subtissular niches^42^, that may be present in the cervix, to be activated by hormones? Moreover, in the absence of inflammation do macrophages and mucins link^43^ to regulate the structure of cervical mucus? The proximity method I describe will identify proteins secreted in the cervical mucus of infection-free ovulating women and serve as background to detect differences of iodinated proteins in the cervical mucus of women with endocrine disorders and help, especially, couples having difficulties in reproduction.

## Figures and Tables

**Figure 1:**
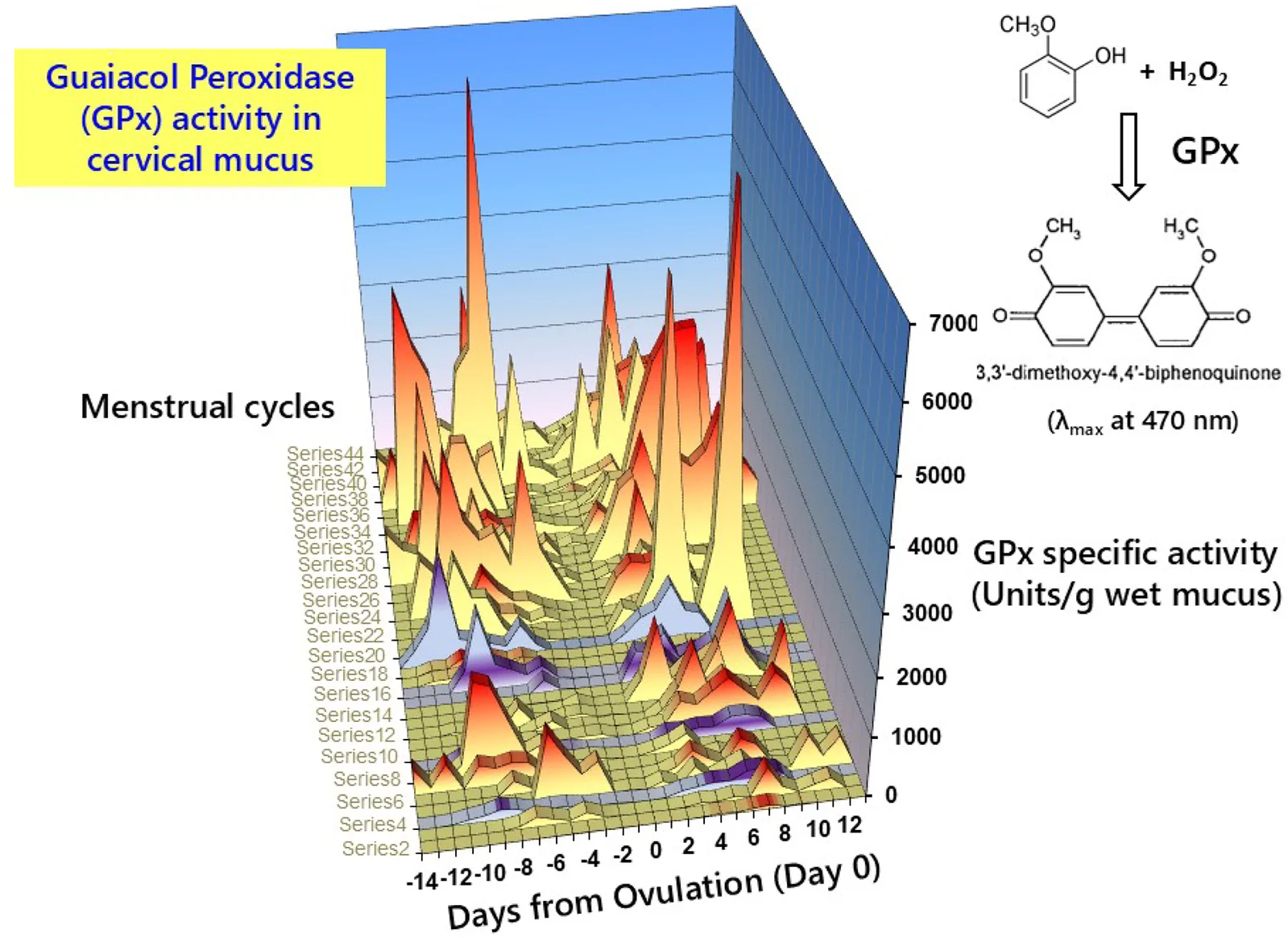
GPx levels in 41 menstrual cycles from different individuals and two from the same individual, shown in blue. All the women were free of infections. Day 0 is the day after the peak of luteinizing hormone (LH) in peripheral blood. GPx is measured spectrophotometrically from the initial linear oxidation of guaiacol (2-methoxyphenol) in the presence of H_2_O_2_ to form the transient product with the suggested structure. Figure 1 was Figure 7 in Tsibris et al^5^ and is redrawn here in a simpler form.

**Figure 2:**
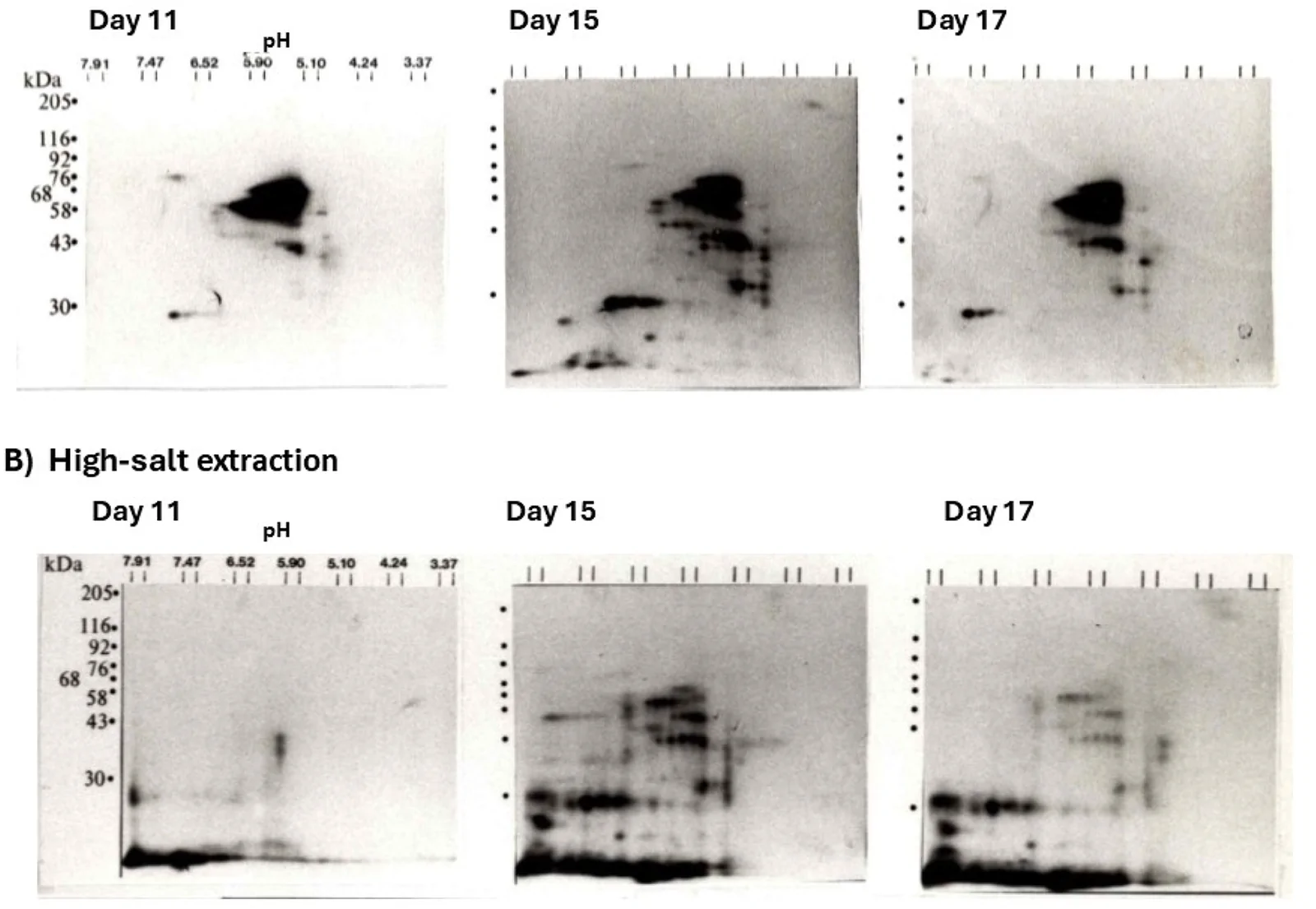
Iodination of three cervical mucus samples from a study volunteer. **A**. Each sample of 4–20 mg was dispersed in 0.3 M Tris-Cl pH 7.2 and 5 mM CaCl_2_ and iodinated, as described in Methods. After dialysis, the dialysates were centrifuged at 100,000*g* for an hour at 8°C and the supernatants, the soluble fraction of cervical mucus, were fractionated by isoelectric focusing and SDS-PAGE and exposed to film; radiograms with optimal band resolution are shown. **B.** The pellets of the 100,000g centrifugation, the particulate fraction of cervical mucus, were extracted overnight in 10 mM Tris-Cl pH 7.5 and 0.5 M CaCl_2_, centrifuged at 100,000*g* for an hour, and the supernatants were dialyzed, fractionated as in **A**, and exposed to film. The GPx specific activity (units/g wet tissue), shown on Figure 8^5^, was 100 on day-11, 0.4 on day-15 and 22 on day-17.

## Data Availability

No additional data are available than provided in this article.
